# 5-Cyclo­hexyl-3-(3-fluoro­phenyl­sulfon­yl)-2-methyl-1-benzofuran

**DOI:** 10.1107/S1600536811011767

**Published:** 2011-04-07

**Authors:** Hong Dae Choi, Pil Ja Seo, Byeng Wha Son, Uk Lee

**Affiliations:** a Department of Chemistry, Dongeui University, San 24 Kaya-dong Busanjin-gu, Busan 614-714, Republic of Korea; b Department of Chemistry, Pukyong National University, 599-1 Daeyeon 3-dong, Nam-gu, Busan 608-737, Republic of Korea

## Abstract

In the title compound, C_21_H_21_FO_3_S, the cyclo­hexyl ring adopts a chair conformation. The 3-fluoro­phenyl ring makes a dihedral angle of 79.15 (4)° with the mean plane of the benzofuran fragment. In the crystal, mol­ecules are linked by weak inter­molecular C—H⋯O hydrogen bonds and C—H⋯π inter­actions.

## Related literature

For the biological activity of benzofuran compounds, see: Aslam *et al.* (2009 ▶); Galal *et al.* (2009 ▶); Khan *et al.* (2005 ▶). For natural products with benzofuran rings, see: Akgul & Anil (2003 ▶); Soekamto *et al.* (2003 ▶). For the structure of 5-cyclo­hexyl-3-(4-fluoro­phenyl­sulfon­yl)-2-methyl-1-benzofuran, see: Choi *et al.* 2011 ▶).
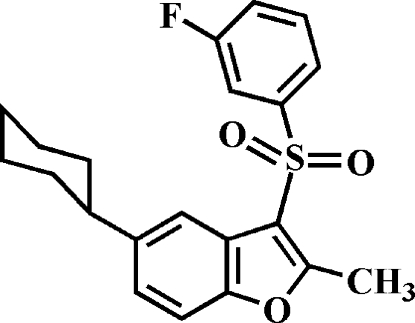


## Experimental

### 

#### Crystal data

C_21_H_21_FO_3_S
*M* *_r_* = 372.44
Triclinic, 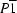

*a* = 9.0456 (2) Å
*b* = 10.1996 (2) Å
*c* = 10.4216 (2) Å
α = 89.915 (1)°
β = 70.461 (1)°
γ = 83.775 (1)°
*V* = 900.18 (3) Å^3^
*Z* = 2
Mo *K*α radiation
μ = 0.21 mm^−1^
*T* = 173 K
0.36 × 0.18 × 0.17 mm

#### Data collection

Bruker SMART APEXII CCD diffractometer
Absorption correction: multi-scan (*SADABS*; Bruker, 2009 ▶) *T*_min_ = 0.603, *T*_max_ = 0.666
16115 measured reflections
4143 independent reflections
3650 reflections with *I* > 2σ(*I*)
*R*_int_ = 0.029

#### Refinement

*R*[*F* ^2^ > 2σ(*F* ^2^)] = 0.041
*wR*(*F* ^2^) = 0.109
*S* = 1.06
4143 reflections
236 parameters
H-atom parameters constrained
Δρ_max_ = 0.28 e Å^−3^
Δρ_min_ = −0.53 e Å^−3^

### 

Data collection: *APEX2* (Bruker, 2009 ▶); cell refinement: *SAINT* (Bruker, 2009 ▶); data reduction: *SAINT*; program(s) used to solve structure: *SHELXS97* (Sheldrick, 2008 ▶); program(s) used to refine structure: *SHELXL97* (Sheldrick, 2008 ▶); molecular graphics: *ORTEP-3* (Farrugia, 1997 ▶) and *DIAMOND* (Brandenburg, 1998 ▶); software used to prepare material for publication: *SHELXL97*.

## Supplementary Material

Crystal structure: contains datablocks global, I. DOI: 10.1107/S1600536811011767/mw2005sup1.cif

Structure factors: contains datablocks I. DOI: 10.1107/S1600536811011767/mw2005Isup2.hkl

Additional supplementary materials:  crystallographic information; 3D view; checkCIF report

## Figures and Tables

**Table 1:** Hydrogen-bond geometry (Å, °) *Cg*1 and *Cg*2 are the centroids of the C1/C2/C7/O1/C8 furan ring and the C2–C7 benzene ring, respectively.

*D*—H⋯*A*	*D*—H	H⋯*A*	*D*⋯*A*	*D*—H⋯*A*
C21—H21⋯O3^i^	0.95	2.38	3.300 (2)	163
C13—H13*A*⋯*Cg*1^ii^	0.99	2.80	3.638 (2)	143
C19—H19⋯*Cg*2^iii^	0.95	2.87	3.660 (2)	141

Symmetry codes: (i) 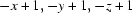; (ii) 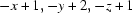; (iii) 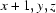.

**Fig. 1.**
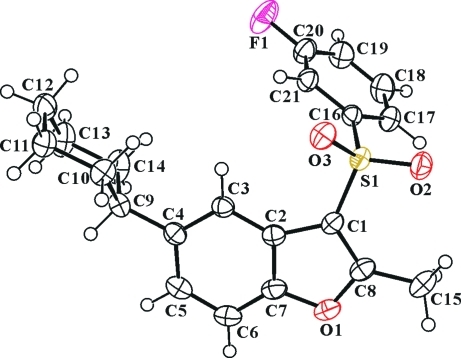
The molecular structure of the title compound with the atom numbering scheme. Displacement ellipsoids are drawn at the 50% probability level. H atoms are presented as a small spheres of arbitrary radius.

**Fig. 2.**
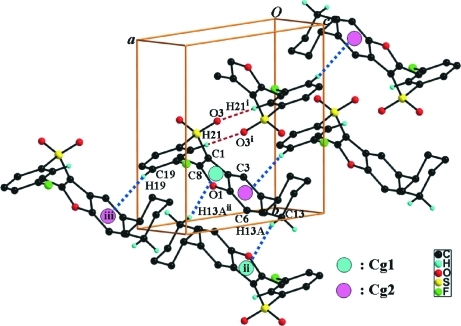
A view of the C—H···O and C—H···π interactions (dotted lines) in the crystal structure of the title compound. [Symmetry codes: (i) - x + 1, - y + 1, - z + 1; (ii) - x + 1, - y + 2, - z + 1; (iii) x + 1, y , z.]

## supplementary crystallographic information

### Comment

Many compounds possessing a benzofuran ring have attracted much attention due to their interesting pharmacological properties such as antibacterial and antifungal, antitumor and antiviral, and antimicrobial activities (Aslam *et al.*, 2009, Galal *et al.*, 2009, Khan *et al.*, 2005). These compounds occur in a wide range of natural products (Akgul & Anil, 2003; Soekamto *et al.*, 2003). As part of our ongoing program of the substituent effect on the solid state structures of 5-cyclohexyl-3-(4-fluorophenylsulfonyl)-2-methyl-1-benzofuran analogues (Choi *et al.*, 2011), we report herein on the crystal structure of the title compound.

In the title molecule (Fig. 1), the benzofuran unit is essentially planar, with a mean deviation of 0.005 (1) Å from the least-squares plane defined by the nine constituent atoms. The 3-fluorophenyl ring makes a dihedral angle of 79.15 (4)°  with the mean plane of the benzofuran ring. The cyclohexyl ring is in the chair form. The crystal packing (Fig. 2) is stabilized by weak intermolecular C—H···O hydrogen bonds (Table 1; C21—H21···O3^i^). The crystal packing (Fig. 2) is further stabilized by intermolecular C—H···π interactions; the first one between a cyclohexyl H atom and the furan ring (Table 1; C13—H13A···Cg1^ii^. Cg1 is the centroid of the  C1/C2/C7/O1/C8 furan ring), and the second one between a 3-fluorophenyl H atom and the benzene ring (Table 1; C19—H19···Cg2^iii^. Cg2 is the centroid of the  C2–C7 benzene ring).

### Experimental

77% 3-chloroperoxybenzoic acid (515 mg, 2.3 mmol) was added in small portions to a stirred solution of 5-cyclohexyl-3-(3-fluorophenylsulfanyl)-2-methyl-1-benzofuran (374 mg, 1.1 mmol) in dichloromethane (40 mL) at 273 K. After being stirred at room temperature for 6h, the mixture was washed with saturated sodium bicarbonate solution and the organic layer was separated, dried over magnesium sulfate, filtered and concentrated at reduced pressure. The residue was purified by column chromatography (hexane-ethyl acetate, 4:1 v/v) to afford the title compound as a colorless solid [yield 71%, m.p. 444–445 K; *R*_f_ = 0.63 (hexane–ethyl acetate, 2:1 v/v)]. Single crystals suitable for X–ray diffraction were prepared by slow evaporation of a solution of the title compound in benzene at room temperature.

### Refinement

All H atoms were positioned geometrically and refined using a riding model, with C—H = 0.95 Å for aryl, 1.00 Å for methine, 0.99Å for methylene and 0.98 Å for methyl H atoms, respectively. *U*_iso_(H) =1.2*U*_eq_(C)  for aryl,  methine and methylene, and 1.5*U*_eq_(C) for methyl H atoms.

### Crystal data

**Table:**

C_21_H_21_FO_3_S	*Z* = 2
*M**_r_* = 372.44	*F*(000) = 392
Triclinic, *P*1	*D*_x_ = 1.374 Mg m^−^^3^
Hall symbol: -P 1	Mo *K*α radiation, λ = 0.71073 Å
*a* = 9.0456 (2) Å	Cell parameters from 7640 reflections
*b* = 10.1996 (2) Å	θ = 2.4–27.5°
*c* = 10.4216 (2) Å	µ = 0.21 mm^−^^1^
α = 89.915 (1)°	*T* = 173 K
β = 70.461 (1)°	Block, colourless
γ = 83.775 (1)°	0.36 × 0.18 × 0.17 mm
*V* = 900.18 (3) Å^3^	

### Data collection

**Table:**

Bruker SMART APEXII CCD diffractometer	4143 independent reflections
Radiation source: rotating anode	3650 reflections with *I* > 2σ(*I*)
graphite multilayer	*R*_int_ = 0.029
Detector resolution: 10.0 pixels mm^-1^	θ_max_ = 27.5°, θ_min_ = 2.0°
φ and ω scans	*h* = −11→11
Absorption correction: multi-scan (*SADABS*; Bruker, 2009)	*k* = −13→13
*T*_min_ = 0.603, *T*_max_ = 0.666	*l* = −13→13
16115 measured reflections	

### Refinement

**Table:**

Refinement on *F*^2^	Primary atom site location: structure-invariant direct methods
Least-squares matrix: full	Secondary atom site location: difference Fourier map
*R*[*F*^2^ > 2σ(*F*^2^)] = 0.041	Hydrogen site location: difference Fourier map
*wR*(*F*^2^) = 0.109	H-atom parameters constrained
*S* = 1.06	*w* = 1/[σ^2^(*F*_o_^2^) + (0.0554*P*)^2^ + 0.2992*P*] where *P* = (*F*_o_^2^ + 2*F*_c_^2^)/3
4143 reflections	(Δ/σ)_max_ = 0.001
236 parameters	Δρ_max_ = 0.28 e Å^−^^3^
0 restraints	Δρ_min_ = −0.53 e Å^−^^3^

### Special details

**Table:**

Geometry. All esds (except the esd in the dihedral angle between two l.s. planes) are estimated using the full covariance matrix. The cell esds are taken into account individually in the estimation of esds in distances, angles and torsion angles; correlations between esds in cell parameters are only used when they are defined by crystal symmetry. An approximate (isotropic) treatment of cell esds is used for estimating esds involving l.s. planes.
Refinement. Refinement of F^2^ against ALL reflections. The weighted R-factor wR and goodness of fit S are based on F^2^, conventional R-factors R are based on F, with F set to zero for negative F^2^. The threshold expression of F^2^ > 2sigma(F^2^) is used only for calculating R-factors(gt) etc. and is not relevant to the choice of reflections for refinement. R-factors based on F^2^ are statistically about twice as large as those based on F, and R- factors based on ALL data will be even larger.

### Fractional atomic coordinates and isotropic or equivalent isotropic displacement parameters (Å^2^)

**Table:**

	*x*	*y*	*z*	*U*_iso_*/*U*_eq_	
S1	0.64474 (4)	0.52983 (3)	0.21026 (3)	0.02707 (11)	
O1	0.46999 (13)	0.83098 (12)	0.05173 (11)	0.0369 (3)	
O2	0.72428 (14)	0.45232 (11)	0.08679 (11)	0.0378 (3)	
O3	0.53423 (13)	0.47240 (11)	0.32307 (11)	0.0339 (2)	
F1	0.85086 (14)	0.65518 (14)	0.58340 (10)	0.0617 (3)	
C1	0.54798 (16)	0.67184 (14)	0.17081 (14)	0.0277 (3)	
C2	0.43810 (16)	0.76821 (14)	0.26820 (14)	0.0270 (3)	
C3	0.37504 (16)	0.78392 (14)	0.40976 (14)	0.0274 (3)	
H3	0.4031	0.7196	0.4661	0.033*	
C4	0.27026 (16)	0.89545 (15)	0.46707 (15)	0.0291 (3)	
C5	0.23043 (18)	0.98894 (16)	0.38158 (17)	0.0367 (3)	
H5	0.1588	1.0647	0.4218	0.044*	
C6	0.29155 (19)	0.97501 (17)	0.24094 (17)	0.0390 (4)	
H6	0.2638	1.0388	0.1840	0.047*	
C7	0.39458 (18)	0.86375 (16)	0.18831 (15)	0.0324 (3)	
C8	0.56287 (18)	0.71427 (16)	0.04348 (15)	0.0325 (3)	
C9	0.20113 (17)	0.91962 (15)	0.61977 (15)	0.0304 (3)	
H9	0.1099	0.9901	0.6381	0.036*	
C10	0.13780 (19)	0.79815 (16)	0.69548 (15)	0.0340 (3)	
H10A	0.0562	0.7692	0.6614	0.041*	
H10B	0.2248	0.7253	0.6766	0.041*	
C11	0.06665 (19)	0.82635 (17)	0.84864 (16)	0.0360 (3)	
H11A	−0.0278	0.8921	0.8684	0.043*	
H11B	0.0327	0.7443	0.8945	0.043*	
C12	0.18420 (19)	0.87831 (18)	0.90463 (17)	0.0399 (4)	
H12A	0.1324	0.9012	1.0030	0.048*	
H12B	0.2729	0.8086	0.8946	0.048*	
C13	0.2477 (2)	0.9990 (2)	0.83106 (18)	0.0487 (5)	
H13A	0.3291	1.0272	0.8657	0.058*	
H13B	0.1610	1.0721	0.8499	0.058*	
C14	0.3196 (2)	0.9707 (2)	0.67729 (18)	0.0461 (4)	
H14A	0.3548	1.0526	0.6317	0.055*	
H14B	0.4134	0.9043	0.6578	0.055*	
C15	0.6550 (2)	0.66339 (19)	−0.09680 (16)	0.0428 (4)	
H15A	0.7559	0.7007	−0.1280	0.064*	
H15B	0.5953	0.6888	−0.1577	0.064*	
H15C	0.6748	0.5670	−0.0975	0.064*	
C16	0.79005 (16)	0.58573 (14)	0.26896 (14)	0.0266 (3)	
C17	0.93127 (18)	0.61397 (16)	0.17467 (15)	0.0355 (3)	
H17	0.9500	0.6034	0.0798	0.043*	
C18	1.04423 (19)	0.65769 (19)	0.22081 (18)	0.0436 (4)	
H18	1.1412	0.6781	0.1572	0.052*	
C19	1.0176 (2)	0.67208 (19)	0.35860 (18)	0.0432 (4)	
H19	1.0953	0.7019	0.3907	0.052*	
C20	0.8763 (2)	0.64229 (18)	0.44828 (16)	0.0388 (4)	
C21	0.75994 (17)	0.59884 (15)	0.40801 (14)	0.0309 (3)	
H21	0.6633	0.5787	0.4723	0.037*	

### Atomic displacement parameters (Å^2^)

**Table:**

	*U*^11^	*U*^22^	*U*^33^	*U*^12^	*U*^13^	*U*^23^
S1	0.03041 (19)	0.02704 (19)	0.02228 (18)	−0.00556 (14)	−0.00621 (14)	0.00027 (13)
O1	0.0414 (6)	0.0446 (6)	0.0301 (5)	−0.0070 (5)	−0.0186 (5)	0.0081 (5)
O2	0.0465 (6)	0.0354 (6)	0.0278 (5)	−0.0020 (5)	−0.0085 (5)	−0.0066 (4)
O3	0.0365 (6)	0.0341 (6)	0.0307 (5)	−0.0126 (5)	−0.0078 (4)	0.0060 (4)
F1	0.0603 (7)	0.1001 (10)	0.0308 (5)	−0.0246 (7)	−0.0187 (5)	−0.0056 (6)
C1	0.0284 (7)	0.0323 (7)	0.0239 (6)	−0.0074 (6)	−0.0094 (5)	0.0019 (5)
C2	0.0246 (6)	0.0290 (7)	0.0302 (7)	−0.0067 (5)	−0.0116 (5)	0.0031 (6)
C3	0.0254 (6)	0.0287 (7)	0.0290 (7)	−0.0041 (5)	−0.0100 (5)	0.0035 (5)
C4	0.0237 (6)	0.0296 (7)	0.0344 (7)	−0.0050 (5)	−0.0098 (6)	0.0013 (6)
C5	0.0327 (8)	0.0319 (8)	0.0467 (9)	0.0008 (6)	−0.0162 (7)	0.0028 (7)
C6	0.0399 (8)	0.0380 (8)	0.0444 (9)	−0.0016 (7)	−0.0219 (7)	0.0108 (7)
C7	0.0309 (7)	0.0395 (8)	0.0316 (7)	−0.0073 (6)	−0.0159 (6)	0.0064 (6)
C8	0.0350 (7)	0.0388 (8)	0.0281 (7)	−0.0110 (6)	−0.0144 (6)	0.0037 (6)
C9	0.0255 (7)	0.0282 (7)	0.0342 (7)	−0.0010 (6)	−0.0065 (6)	−0.0020 (6)
C10	0.0373 (8)	0.0332 (8)	0.0333 (8)	−0.0102 (6)	−0.0125 (6)	−0.0004 (6)
C11	0.0349 (8)	0.0391 (8)	0.0328 (8)	−0.0079 (7)	−0.0088 (6)	−0.0002 (6)
C12	0.0361 (8)	0.0484 (10)	0.0347 (8)	−0.0009 (7)	−0.0125 (7)	−0.0089 (7)
C13	0.0472 (10)	0.0541 (11)	0.0421 (9)	−0.0215 (9)	−0.0066 (8)	−0.0154 (8)
C14	0.0394 (9)	0.0550 (11)	0.0409 (9)	−0.0218 (8)	−0.0045 (7)	−0.0107 (8)
C15	0.0525 (10)	0.0531 (10)	0.0249 (7)	−0.0121 (8)	−0.0138 (7)	0.0030 (7)
C16	0.0271 (6)	0.0255 (7)	0.0250 (6)	−0.0021 (5)	−0.0063 (5)	0.0016 (5)
C17	0.0341 (8)	0.0409 (9)	0.0268 (7)	−0.0063 (7)	−0.0035 (6)	0.0013 (6)
C18	0.0309 (8)	0.0529 (10)	0.0412 (9)	−0.0133 (7)	−0.0017 (7)	0.0009 (8)
C19	0.0335 (8)	0.0515 (10)	0.0466 (9)	−0.0104 (7)	−0.0144 (7)	−0.0037 (8)
C20	0.0402 (8)	0.0473 (9)	0.0298 (8)	−0.0062 (7)	−0.0127 (7)	−0.0027 (7)
C21	0.0295 (7)	0.0363 (8)	0.0245 (7)	−0.0054 (6)	−0.0055 (5)	0.0016 (6)

### Geometric parameters (Å, °)

**Table:**

S1—O2	1.4349 (11)	C10—H10B	0.9900
S1—O3	1.4365 (11)	C11—C12	1.513 (2)
S1—C1	1.7312 (15)	C11—H11A	0.9900
S1—C16	1.7687 (14)	C11—H11B	0.9900
O1—C8	1.3658 (19)	C12—C13	1.513 (3)
O1—C7	1.3804 (18)	C12—H12A	0.9900
F1—C20	1.3529 (18)	C12—H12B	0.9900
C1—C8	1.363 (2)	C13—C14	1.529 (2)
C1—C2	1.449 (2)	C13—H13A	0.9900
C2—C7	1.390 (2)	C13—H13B	0.9900
C2—C3	1.3948 (19)	C14—H14A	0.9900
C3—C4	1.392 (2)	C14—H14B	0.9900
C3—H3	0.9500	C15—H15A	0.9800
C4—C5	1.404 (2)	C15—H15B	0.9800
C4—C9	1.512 (2)	C15—H15C	0.9800
C5—C6	1.384 (2)	C16—C17	1.386 (2)
C5—H5	0.9500	C16—C21	1.3859 (19)
C6—C7	1.374 (2)	C17—C18	1.379 (2)
C6—H6	0.9500	C17—H17	0.9500
C8—C15	1.481 (2)	C18—C19	1.380 (2)
C9—C14	1.527 (2)	C18—H18	0.9500
C9—C10	1.528 (2)	C19—C20	1.372 (2)
C9—H9	1.0000	C19—H19	0.9500
C10—C11	1.523 (2)	C20—C21	1.371 (2)
C10—H10A	0.9900	C21—H21	0.9500
			
O2—S1—O3	119.54 (7)	C12—C11—H11B	109.4
O2—S1—C1	108.36 (7)	C10—C11—H11B	109.4
O3—S1—C1	107.84 (7)	H11A—C11—H11B	108.0
O2—S1—C16	107.91 (7)	C13—C12—C11	111.25 (14)
O3—S1—C16	107.44 (6)	C13—C12—H12A	109.4
C1—S1—C16	104.81 (7)	C11—C12—H12A	109.4
C8—O1—C7	107.21 (11)	C13—C12—H12B	109.4
C8—C1—C2	107.75 (13)	C11—C12—H12B	109.4
C8—C1—S1	126.44 (12)	H12A—C12—H12B	108.0
C2—C1—S1	125.81 (11)	C12—C13—C14	111.28 (14)
C7—C2—C3	119.28 (13)	C12—C13—H13A	109.4
C7—C2—C1	104.39 (13)	C14—C13—H13A	109.4
C3—C2—C1	136.33 (13)	C12—C13—H13B	109.4
C4—C3—C2	118.89 (13)	C14—C13—H13B	109.4
C4—C3—H3	120.6	H13A—C13—H13B	108.0
C2—C3—H3	120.6	C9—C14—C13	111.48 (13)
C3—C4—C5	119.47 (14)	C9—C14—H14A	109.3
C3—C4—C9	121.33 (13)	C13—C14—H14A	109.3
C5—C4—C9	119.19 (13)	C9—C14—H14B	109.3
C6—C5—C4	122.50 (15)	C13—C14—H14B	109.3
C6—C5—H5	118.8	H14A—C14—H14B	108.0
C4—C5—H5	118.8	C8—C15—H15A	109.5
C7—C6—C5	116.30 (14)	C8—C15—H15B	109.5
C7—C6—H6	121.9	H15A—C15—H15B	109.5
C5—C6—H6	121.9	C8—C15—H15C	109.5
C6—C7—O1	125.90 (14)	H15A—C15—H15C	109.5
C6—C7—C2	123.57 (15)	H15B—C15—H15C	109.5
O1—C7—C2	110.54 (13)	C17—C16—C21	121.89 (14)
C1—C8—O1	110.12 (13)	C17—C16—S1	119.07 (11)
C1—C8—C15	134.83 (15)	C21—C16—S1	119.03 (11)
O1—C8—C15	115.05 (13)	C18—C17—C16	118.91 (14)
C4—C9—C14	111.66 (12)	C18—C17—H17	120.5
C4—C9—C10	113.05 (12)	C16—C17—H17	120.5
C14—C9—C10	109.95 (13)	C17—C18—C19	120.57 (15)
C4—C9—H9	107.3	C17—C18—H18	119.7
C14—C9—H9	107.3	C19—C18—H18	119.7
C10—C9—H9	107.3	C20—C19—C18	118.49 (15)
C11—C10—C9	111.75 (13)	C20—C19—H19	120.8
C11—C10—H10A	109.3	C18—C19—H19	120.8
C9—C10—H10A	109.3	F1—C20—C21	118.15 (15)
C11—C10—H10B	109.3	F1—C20—C19	118.49 (15)
C9—C10—H10B	109.3	C21—C20—C19	123.35 (15)
H10A—C10—H10B	107.9	C20—C21—C16	116.78 (14)
C12—C11—C10	111.36 (13)	C20—C21—H21	121.6
C12—C11—H11A	109.4	C16—C21—H21	121.6
C10—C11—H11A	109.4		
			
O2—S1—C1—C8	−9.28 (16)	C7—O1—C8—C15	−179.52 (13)
O3—S1—C1—C8	−140.01 (13)	C3—C4—C9—C14	77.39 (18)
C16—S1—C1—C8	105.74 (14)	C5—C4—C9—C14	−101.21 (17)
O2—S1—C1—C2	171.80 (12)	C3—C4—C9—C10	−47.21 (18)
O3—S1—C1—C2	41.07 (14)	C5—C4—C9—C10	134.19 (15)
C16—S1—C1—C2	−73.18 (13)	C4—C9—C10—C11	−179.06 (12)
C8—C1—C2—C7	0.06 (16)	C14—C9—C10—C11	55.41 (17)
S1—C1—C2—C7	179.14 (11)	C9—C10—C11—C12	−55.78 (18)
C8—C1—C2—C3	−179.27 (16)	C10—C11—C12—C13	55.33 (18)
S1—C1—C2—C3	−0.2 (2)	C11—C12—C13—C14	−55.4 (2)
C7—C2—C3—C4	−0.2 (2)	C4—C9—C14—C13	178.28 (15)
C1—C2—C3—C4	179.09 (15)	C10—C9—C14—C13	−55.41 (19)
C2—C3—C4—C5	0.1 (2)	C12—C13—C14—C9	56.0 (2)
C2—C3—C4—C9	−178.48 (12)	O2—S1—C16—C17	33.39 (14)
C3—C4—C5—C6	0.0 (2)	O3—S1—C16—C17	163.52 (12)
C9—C4—C5—C6	178.61 (14)	C1—S1—C16—C17	−81.94 (13)
C4—C5—C6—C7	0.0 (2)	O2—S1—C16—C21	−146.01 (12)
C5—C6—C7—O1	−179.39 (14)	O3—S1—C16—C21	−15.88 (13)
C5—C6—C7—C2	0.0 (2)	C1—S1—C16—C21	98.66 (12)
C8—O1—C7—C6	179.21 (15)	C21—C16—C17—C18	−0.7 (2)
C8—O1—C7—C2	−0.24 (16)	S1—C16—C17—C18	179.92 (13)
C3—C2—C7—C6	0.1 (2)	C16—C17—C18—C19	0.5 (3)
C1—C2—C7—C6	−179.35 (15)	C17—C18—C19—C20	−0.2 (3)
C3—C2—C7—O1	179.58 (12)	C18—C19—C20—F1	179.37 (17)
C1—C2—C7—O1	0.11 (16)	C18—C19—C20—C21	0.0 (3)
C2—C1—C8—O1	−0.20 (17)	F1—C20—C21—C16	−179.53 (14)
S1—C1—C8—O1	−179.28 (10)	C19—C20—C21—C16	−0.2 (3)
C2—C1—C8—C15	179.53 (17)	C17—C16—C21—C20	0.5 (2)
S1—C1—C8—C15	0.5 (3)	S1—C16—C21—C20	179.91 (12)
C7—O1—C8—C1	0.27 (16)		

### Hydrogen-bond geometry (Å, °)

**Table:**

Cg1 and Cg2 are the centroids of the C1/C2/C7/O1/C8 furan ring and the C2–C7 benzene ring, respectively.

**Table:**

*D*—H···*A*	*D*—H	H···*A*	*D*···*A*	*D*—H···*A*
C21—H21···O3^i^	0.95	2.38	3.300 (2)	163.
C13—H13A···Cg1^ii^	0.99	2.80	3.638 (2)	143.
C19—H19···Cg2^iii^	0.95	2.87	3.660 (2)	141.

Symmetry codes: (i) −*x*+1, −*y*+1, −*z*+1; (ii) −*x*+1, −*y*+2, −*z*+1; (iii) *x*+1, *y*, *z*.
